# KEGGconverter: a tool for the in-silico modelling of metabolic networks of the KEGG Pathways database

**DOI:** 10.1186/1471-2105-10-324

**Published:** 2009-10-08

**Authors:** Konstantinos Moutselos, Ioannis Kanaris, Aristotelis Chatziioannou, Ilias Maglogiannis, Fragiskos N Kolisis

**Affiliations:** 1 Department of Informatics with Applications in Biomedicine, University of Central Greece. Papasiopoulou 2-4, 35100, Lamia, Greece; 2 Department of Information and Communication Systems Engineering, University of the Aegean, 83200, Karlovasi, Samos, Greece; 3 Metabolic Engineering and Bioinformatics Group, Institute of Biological Research and Biotechnology, National Hellenic Research Foundation, Vassileos Konstantinou 48, 11635, Athens, Greece

## Abstract

**Background:**

The KEGG Pathway database is a valuable collection of metabolic pathway maps. Nevertheless, the production of simulation capable metabolic networks from KEGG Pathway data is a challenging complicated work, regardless the already developed tools for this scope. Originally used for illustration purposes, KEGG Pathways through KGML (KEGG Markup Language) files, can provide complete reaction sets and introduce species versioning, which offers advantages for the scope of cellular metabolism simulation modelling. In this project, KEGGconverter is described, implemented also as a web-based application, which uses as source KGML files, in order to construct integrated pathway SBML models fully functional for simulation purposes.

**Results:**

A case study of the integration of six human metabolic pathways from KEGG depicts the ability of KEGGconverter to automatically produce merged and converted to SBML fully functional pathway models, enhanced with default kinetics. The suitability of the developed tool is demonstrated through a comparison with other state-of-the art relevant software tools for the same data fusion and conversion tasks, thus illustrating the problems and the relevant workflows. Moreover, KEGGconverter permits the inclusion of additional reactions in the resulting model which represent flux cross-talk with neighbouring pathways, providing in this way improved simulative accuracy. These additional reactions are introduced by exploiting relevant semantic information for the elements of the KEGG Pathways database. The architecture and functionalities of the web-based application are presented.

**Conclusion:**

KEGGconverter is capable of producing integrated analogues of metabolic pathways appropriate for simulation tasks, by inputting only KGML files. The web application acts as a user friendly shell which transparently enables the automated biochemically correct pathway merging, conversion to SBML format, proper renaming of the species, and insertion of default kinetic properties for the pertaining reactions. The tool is available at:

## Background

The Kyoto Encyclopedia of Genes and Genomes (KEGG)[1] PATHWAY database is a valuable comprehensive collection of manually curated pathway maps for metabolism, genetic information processing and other functions. Access to its data is free for academic users and is enabled through various options like: the KEGG ftp site (flat files) [2] in combination with KEGG2SBML[3], by accessing already converted models to SBML[4], through the KEGG API[5], or through KEGG KGML [6]. Nevertheless, the production of simulation capable metabolic networks from KEGG data, in order to run in-silico reaction models is a complicated work, regardless the numerous tools, ostensibly distributed for this task. The in-silico construction of detailed reaction pathways, in particular of specific organisms, entails many steps. Moreover the use of different software tools for these steps, subjects this process more difficult and time consuming, as it requires compatibility among them.

An example of the intricacies involved in the selection of the proper data source, when attempting to integrate KEGG metabolic pathways, is the following:

• already converted SBML[7] models from [4] are usually outdated,

• the use of KEGG API for this scope, requires extra programming,

• handling the flat files is unwieldy,

• the use of KEGG2SBML tool for their conversion to the SBML format has its own drawbacks, since it is operating in Unix-like platforms only. At the same time, downloading the relevant to the conversion flat files, requires the knowledge of the exact url address and path, to the respective directories of the KEGG ftp server.

The tool KEGG2SBML was developed in order to create SBML models, by parsing KEGG data found in the KEGG LIGAND database, under the ERATO-SORST Kitano Symbiotic Systems Project [8]. This tool was implemented using the Perl programming language in 2004 and managed to transform accurately about 93%[9] of the total metabolic pathway models, available in the database, at that time. A new version (1.5.0) released in 2008, is capable of exporting SBML models in both Level1 and Level2 format. It also supports the annotation scheme of CellDesigner[10], which retains the original visual layout of KEGG models. CellDesigner is a powerful tool for designing and simulating SBML pathway models, compliant with the Systems Biology Workbench (SBW)[11], with an user-friendly graphical interface. This enables distinct representation of various entity types, through the use of metadata, imported as annotations into standard SBML models.

KEGG Markup Language (KGML) files provide graph information that can be used to computationally reproduce and manipulate KEGG pathway maps [12]. Nevertheless, two major issues should be stressed here, concerning the transformation of KEGG metabolic pathways to biochemical models, appropriate for in-silico functional simulation of various aspects of the cellular physiology. First of all KGML, which is an XML based format specific for data representation, does not include kinetic information (rate laws) for the pertaining reactions. It is oriented in providing graphic illustrations of the pathways, mainly appropriate for visualization purposes, but can also provide graph operation capabilities, through the use of appropriate software tools, like KGML-ED [13] and KEGGgraph [14]. For dynamic in-silico modelling efforts though, a limitation arises regarding the extent of exploitation of this valuable knowledge source, as KGML model files are inappropriate for simulation purposes. This holds even though they form a compendium of pathways, fully compliant with the traditional biochemical functional characterization. KGML format cannot carry information regarding the stoichiometry and the kinetic properties of the reaction equations of the illustrated pathway, an essential prerequisite for the build-up of a simulated biochemical reaction model. Manually adding the kinetic laws in biochemical models represents a time exhaustive effort, depending on the size of the pathways to be modelled, in terms of encompassed reactions, substrates and modifiers.

Secondly the orientation of the KGML models, residing in the KEGG pathway database, mainly for visualization purposes, incurs serious pitfalls, with respect to the biochemical consistency of the described models for simulation tasks. In order to attain a nicer graphical layout, in terms of clarity of the delineation of the graph, many compounds and reactions are included more than once in the same pathway. This poses a problem regarding the biochemical consistency of the model, given the fact that all reactions take place in the same cell compartment, unless modelling a multi-compartmental model. Consequently this results in erroneous stoichiometries for the respective reaction network. Also the existence of ambiguities regarding the appellations of the compounds included in a model, which represent the result of unresolved inconsistencies in the nomenclatures used, results in problematic definition of the quantitative characteristics of the participating metabolic pools (i.e. overall concentration of an enzyme or compound) in the model. This incurs additional flaws and increases the complexity of the model. Unfortunately, all these problems remain unresolved in the KEGG2SBML approach, where the thus transformed models are still simple mappings of the KEGG database to static SBML models, with no addition of kinetic properties and not any correction on their descriptive problems.

Furthermore, when scrutinizing the KGML and SBML versions of KEGG residing metabolic pathways, transformed through KEGG2SBML, differences are found between them, with respect to the composition and the stoichiometry of the resulting reaction networks. Indicatively, the SBML version includes fewer reactions than the KGML model and for each reaction it incorporates species such as: H2O, H+, NADH, NAD+, ATP, ADP, CO2, CoA. For simulation purposes though, elimination from the network of such compounds, which participate in numerous reactions simultaneously and which pools are considered abundant, is an acceptable model simplification practice [15]. Therefore, original KGML files are considered as the preferred data source for transformation purposes, for the accurate integration of KEGG metabolic networks.

In this work, we implemented the KEGGconverter tool, which executes and integrates -for the first time- the following procedures: pathway merging, conversion to SBML format, proper renaming of the species and addition of default kinetic equations, using as sole input the set of selected KGML KEGG pathway file. The resulted output is an SBML model, which can be tested in a simulation environment, and can be used as a reference point for further improvement and accurate tuning of the model.

## Implementation

**Figure 1:**
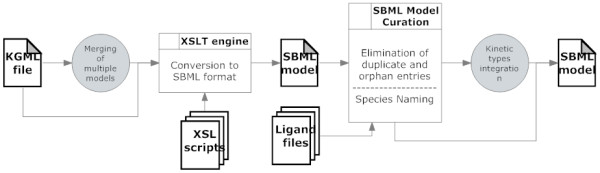
**KEGGconverter: KGML to SBML conversion stages**. KEGGconverter is a de novo developed software application. It implements a novel algorithmic workflow, which aims at the proper transformation of the set of original KGML files, in order to be fused together in one SBML file, by using a combination of XSL and Java procedures.

KEGGconverter is a de novo developed software application. It implements a novel algorithmic workflow (Figure 1), which aims at the proper transformation of the set of original KGML files, in order to be fused together in one SBML file, by using a combination of XSL and Java procedures. The three distinct phases are:

• KGML merging,

• conversion to SBML,

• and the addition of kinetics.

### Merging KGML pathways

In order to enable the fusion of many interrelated KEGG pathways, for the scope of building system-level models, an algorithm was implemented, to merge selected KGML models. This entails the concatenation of all entries, relations and reaction elements from the selected pathways, into a single file. During this concatenation, the 'id' attribute values from the <entry> elements and the 'entry1-2' attribute values form the <relation> elements are modified, so as to incorporate an indication of the file number, they have originated from. Additionally, during the concatenation process, <relation> elements with target pathways that are to be merged, are removed. Thus, discrepancies in the connecting arrows of the resulting integrated pathway are avoided. These arrows will no longer point (map links) to the already incorporated pathways, in the model.

### Conversion to the SBML format

Subsequently the conversion of the single file, derived from the previous step to a SBML file, is handled. This is a two stage process, which entails a combination of XSL transformations and then DOM processing through Java programming. This procedure is depicted at Figure 1.

Firstly, the elements of <entry> group in the KGML file are forming the <species> group in the SBML format. This group includes compounds, genes or enzymes and neighbouring pathways. Accordingly, the <reaction> elements in SBML are created, using information residing in both <reaction> and <relation> elements of the KGML input file. There, it is specified how the compounds of the network interconnect with each other. Specifically, the <reaction> elements in KGML define the reactants and products, while modifiers are defined in <relation> elements, which link to certain enzymes in the <entry> group, as well as to anchor points of the neighbouring pathways.

A core idea in this stage, is the conversion of the <relation> KGML elements, which point to other pathways, into additional SBML reactions. In this way, the map link information of the KGML pathway files is retained during this process. If the map link points at an included pathway, the redundant SBML 'pathway' <species> entry will be eliminated, at the second stage of the conversion. Otherwise, these entries will be retained at the SBML file. They will be considered as conventional reactions in the final simulation, indicating group reaction flows towards neighboring pathways. In this way, more accurate constraining is attained, with respect to the putative biochemical cross-talks of the integrated pathway model with neighboring pathways, which have not been incorporated in the final model. By having distinct 'id' and 'name' values from the rest of KEGG reactions, these reactions are fully identifiable and can thus be easily modified, erased or included fully or partially to the model of the resulting SBML file. Optionally, an XSL file has been created, which removes completely these reactions, if the user wishes so.

After the initial XSL transformation stage, the resulting file is already complying with the SBML format, and can be read and edited by every tool supporting this format.

However, there are still two main lingering problems, with respect to the thus created SBML models, namely the nomenclature adopted for the species, and the redundancy of both species and reactions. By utilizing the source information from KEGG database, all model species are named according to their enzyme commission numbers (EC), i.e. 'ec:2.3.1.180' and 'cpd:C00229', instead of obtaining informative biochemical names. This clearly renders them illegible and incomprehensive. Another important issue is that of species and relation entries redundancy. Target is the neat and at the same time biochemically consistent, graphical representation of the KEGG pathways. Due to the previous merging step though, multiple pathway xml files are integrated in one file. Consequently, their particular species and relation entries are added in the final file, without been checked, whether they refer to the same species (biochemical compounds) or relations (reactions), which take place within the same cellular compartment. As a result, multiple graphical instances (nodes for species and arrows for relations) of the same elements are encountered, within the derived xml file. In order to eliminate these problems, the model is further processed through the use of Java programmed procedures, which utilize the Xerces XML parser and the DOM. The curation of the models entails two distinct steps:

### Elimination of duplicate and orphan entries

**Figure 2:**
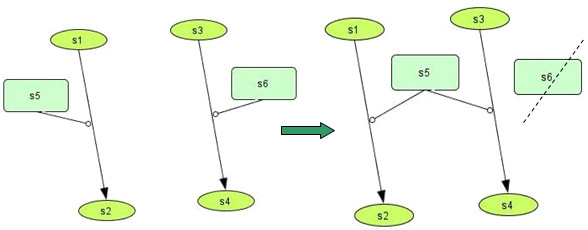
**Duplicates and orphans elimination**. a. Initial state of two reactions with the modifiers s5 and s6 having different ids but representing the same entity (by having the same name). b. The final state, where s5 is defined as modifier for the second reaction too, and s6 is erased.

At this stage, all species are checked based on their KEGG name. If the same name is found in more than one species, then all these species obtain as 'id' value the first value found. An example can be seen at Figure 2, where an illustration of two different reactions is given, each having one reactant, one product and one modifier. In the case that the modifier s5 is the same with the s6, the first will replace the second in its reaction.

The same method is applied also for duplicate <reaction> elements, where all attributes - such as name, reactants, products and modifiers - are the same. This usually happens, when fusing more than one neighbouring pathway models, which have overlapping sections.

Performing this substitution however, deprives some species completely of their reference to any reaction. These "orphan" nodes are eliminated, together with other already "orphan" nodes from the initial model (often in KEGG pathways appear metabolites and enzymes, not connected with other entities of the presented reaction network). However, if for any reason, the user wants to preserve these orphan nodes, this step can be overridden either by executing the appropriate commands in the command line tool, or by ticking the respective box in the web application. For a description of the commands of the command line version, one can either check the relevant supplementary material (Additional file 1) or visit the relevant link (Download Tool) in the application's web page, where the respective commands are described.

### Naming <species>

**Figure 3:**
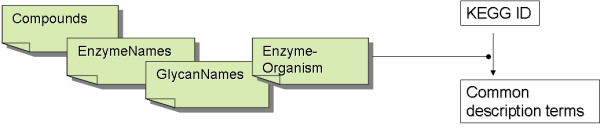
**Parsed tab files for the name decoding step of <species>**. The solution adopted here was instead, that of parsing selected flat files, from the KEGG database and creating four tab delimited files with all the necessary information.

After the removal of all unnecessary entries from the model, renaming of the species, according to the KEGG database nomenclature, follows. Although it is possible to retrieve the name of species by submitting queries directly to the dbget system (e.g. for instance for the ec:1.3.1.9 name ), the solution adopted here was instead, that of parsing selected flat files, from the KEGG database and creating four tab delimited files with all necessary information (Figure 3). The reason for that was the time profit thus attained, as the time needed to parse a tab delimited file and load it into memory, is far less than that needed to submit massively, internet queries. The four tab delimited files are: Compounds.tab, EnzymeNames.tab, GlycanNames.tab and Enzyme-Organism.tab.

During this step, the values of the 'name' attribute of the <species> elements are being searched through the appropriate .tab file for a match. In order to decide, if the ID in question is a compound, enzyme, glycan or gene, the prefix of the ID is examined. If the name starts with the prefix "cpd:" it refers to a compound, "glycan:" is for a Glycan, "ec:" refers to Enzyme and if it has organism id followed by ":" it's a gene. For example "hsa:893" is the human ortholog gene with index 893, (homo sapiens = hsa). In reference pathways, reactions are catalyzed by enzymes, while in organism specific pathways, genes are shown as reaction modifiers. Using the Enzyme-Organism.tab, we can correlate gene ortholog for the specific organism with the related encoded enzyme and both are assigned as a name to the entity. In many cases though, an enzyme is encoded by more than one gene, but only the first of them found in the database, is utilized in this renaming procedure, so as to avoid extremely long name tags.

After matching each id with the respective nomenclature found in the KEGG tab delimited files, a tag name is created and imported in the model. This label represents a comprehensible name for the user, when the model is opened from any SBML editing tool. In order to be information rich, final names also include the original KEGG Ids. Specifically in the case of compounds, the biological name is assigned together with the original ID in brackets (e.g. for the compound cpd:C01236 final name is "D-Glucono-1,5-lactone 6-phosphate [C01236]"). In the case of enzyme encoding genes (functionally linked with catalytic reactions), the resulting name includes both the name of the relevant enzyme and EC number, as well as those of the related protein encoding gene (e.g for the gene AT1G24360 the final name is "3-oxoacyl- [acyl-carrier-protein] reductase [1.1.1.100] [ath:AT1G24360]").

### Massive Introduction of Kinetic Information

The final phase to the derivation of a fully capable pathway model for dynamic simulation purposes, is the addition of the layer of kinetic equations. For this purpose, we used a proprietary kinetic library, fruit of our previous development effort [16], as a plug-in on SBMLeditor[17]. This library includes 17 types of kinetic equations for reversible reactions and 15 for non reversible ones, but is customized to include also user-defined kinetic mechanisms. By exploiting the reaction type (reversible or non-reversible) and the stoichiometric relations of each reaction's reactants, products and modifiers, KEGGconverter enables the automated introduction of 4 case-specific default kinetic mechanisms in the model, according to the following rules:

• If a reaction is of 1-1-1 type (which means: 1 reactant, 1 product, 1 modifier) then the hyperbolic modifier rate-law is being added. If the reaction is reversible, the reversible hyperbolic modifier equation is being added.

• For all remaining types of reactions, the mass action rate law is being added (appropriately modified for the reversible or irreversible cases). If there are modifiers in the reaction, they are retained in the SBML derived file, although the mass action mechanism does not foresee modifiers, so that no loss of information occurs. In this case, it is left upon the user to decide later, how to tackle each specific case.

The implemented tool is freely available to the academic community, both as a web application as well as a java .jar command line tool. The user can select the KEGG pathways he/she wishes to process and may apply several of the following options:

• merging to a single KGML model

• conversion to SBML format

• conversion to SBML plus the addition of default kinetic equations.

### Web Application

**Figure 4:**
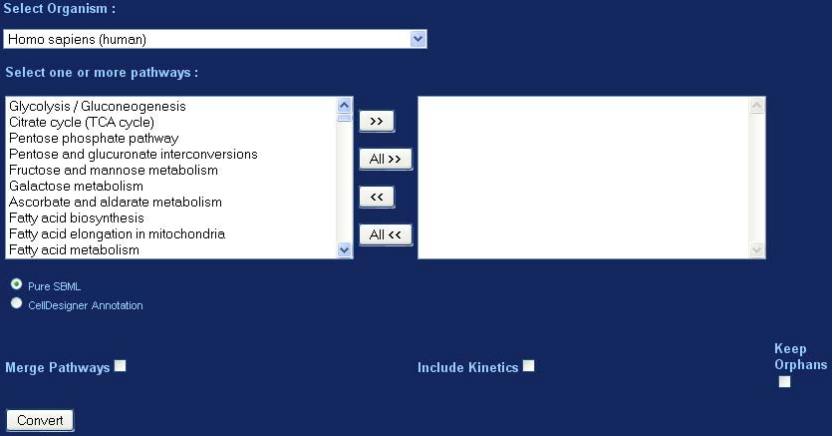
**Web based Tool Interface**. A screen grab depicting the interface used in the study.

Regarding the Web based version of KEGGconverter, users can access it through the homepage of the project. A user-friendly shell is wrapping the inner algorithms of the application. This ensures easy access and manipulations to its content, as the whole process is transparently and seamlessly performed. On the introductory page, the user can set all the necessary properties for performing the conversion (Figure 4).

In the web version of KEGGconverter, the inner logic of the tool is implemented through the use of listboxes and buttons, which enact appropriate PHP scripts and execute specific scenarios. The selection of input KGML files is performed through the use of three list boxes. The first one, concerns the organisms, which pathways are to be studied. All organisms are listed following the official KEGG taxonomy. Next, through the use of two list boxes, the user can create a list of pathways to be fused and converted, by selecting those from the first box and importing them to the second. After the selection step, the parameters for the type of conversion that will be performed need to be set. By using a set of radio buttons, the conversion to either pure-SBML or Celldesigner-SBML annotated model is defined. The latter encloses more detailed information (kinetic information plus characterizations of the compound type as protein, gene or other biochemical types), appropriate for a "vertical" biochemical description, which spans the distance from the genotype to the cellular phenotype. Pathway fusion and/or kinetic laws incorporation to the final model is implemented by ticking two specific checkboxes. Having set all aforementioned parameters, by clicking the Convert button, the requested operation is performed. When the conversion is completed, the user is navigated to the results screen. In this screen, a text box with the label "Executed Command(s):" lists the exact series of commands executed, in order to perform this conversion. A second textbox labelled "Conversion Log:" lists the output of the tool during runtime. This is useful for debugging purposes, as it provides users with the list of commands that can be used as scripts, for the command line version of the tool. Finally, a link for downloading a zip file with all converted models, can be found at the bottom of the page.

Through this convenient web shell, the fusion, correction, kinetic properties incorporation and conversion tasks are performed at a snap. The user needs not download any software or dispose a computer with upgraded capacities in terms of computational power, performance of memory or storage. This tool wraps seamlessly the java implementation tool described earlier. Also, the task of software maintenance or upgrade, in order to keep up with recent developments, with respect to file formats compatibility is averted. The four tab delimited files depicted in Figure 3 are automatically updated weekly, in order to be in full compliance with the latest releases of the KEGG database. In this way, no particular knowledge, as regards the use of scripting commands for the tuning of the routines, is required. The web application automatically retrieves the specified KGML files directly from the KEGG repository and converts them in the background. This web tool is deployed on a Scientific Linux 3.0 based Apache server using PHP v4.3.9 for design, data retrieval and program execution.

## Results & Discussion

In order to highlight the application of KEGGconverter, a case study is presented concerning the integration of 6 KEGG pathways, related to the central metabolism of the Homo sapiens Organism (human), to a single model:

• Glycolysis/Gluconeogenesis - map00010

• Citrate cycle (TCA cycle) - map00020

• Pentose phosphate pathway - map00030

• Fatty acid biosynthesis - map00061

• Fatty acid metabolism - map00071

• Urea cycle and metabolism of amino groups - map00220.

Next, we implement a comparative analysis of the performance of KEGGconverter, with two specific workflows, exploiting other state of the art tools, for the implementation of analogous tasks.

**Figure 5:**
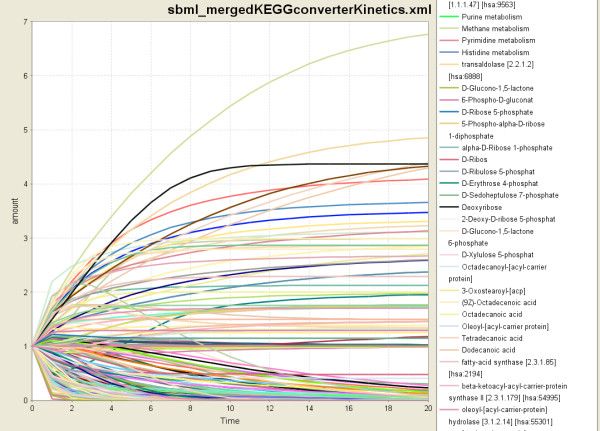
**The case study pathway simulated in CellDesigner**. In the legend of the Figure, only part of the participated species is shown, due to its enormous number. x-axis corresponds to time (unit secs here) whereas y-axis corresponds to species concentrations (mol/L). The number of species of the resulted model is 266, including the neighbouring pathways represented as distinct species. For example: the purine, methane, pyrimidine and histidine metabolisms which are shown at the top of the legend. These neighbouring pathways, among others which are not apparent in the legend, take part in the simulation of our resulted model, and offer additional information for the direction of the metabolic flow of the integrated reaction network.

Regarding KEGGconverter, the necessary data acquisition procedure that must be followed, is the selection of the 6 pathways and their transfer to the input directory of KEGGconverter [See additional file 2: 6 kgml files as the input data for this case study, and the produced models]. The user selects whether default kinetics is to be added by using the "makeKinetics" or "justConvert" command parameter to perform just the transformation to SBML, and the conversion begins. At the end of the conversion the integrated model is transferred to the output directory. The model is ready for inspection as well as to serve as a base for subsequent experimental simulations in an appropriate software environment. A simulation of the resulted model having default initial concentration values for the species is illustrated in Figure 5.

In comparison to this automated procedure, we enlist two attempts to conclude to a similar outcome, either by using KGML-ED which is capable of importing KGML files, or by a combination of other tools starting with KEGG2SBML for the production of SBML converted models.

### The KGML-ED case

**Figure 6:**
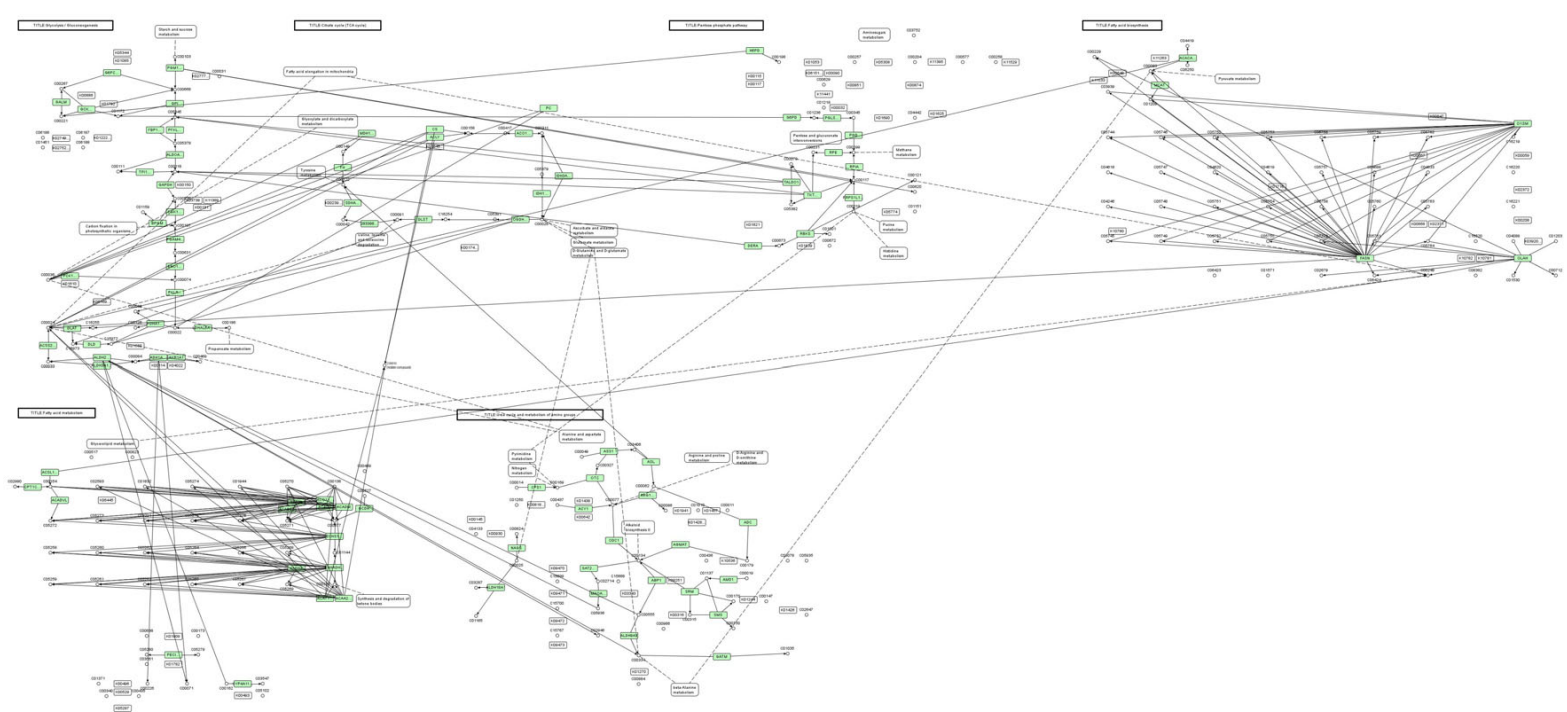
**The case study integrated network using KGML-ED**. A graph displaying the case study integrated network using KGML-ED.

Using KGML-ED[13] which can be run as a Java Web-Start application, we can import the six KGML files and merge the model to an integrated network. Selecting 'Condense into single entities' the duplicate elements are removed and the resulting pathway can be saved as a file in KGML format. At Figure 6, the graph of the integrated network using KGML-ED is shown. The next step was to use KEGG2SBML, which allegedly according to [3] could use and parse KGML files to SBML. Noteworthy, the recent version of the tool does not make use at all of KGML files as input, but .coord or .conf flat files from the KEGG Metabolic Pathway database and the LIGAND database. So this analysis pipeline could not be further implemented for the specific simulation task.

Here it should be mentioned, that tools such as VisANT[18] and VANTED[19] have also the ability to load KEGG Pathways, but they cannot convert the resulted networks to SBML.

### KEGG2SBML - semanticSBML/mergeSBML - SBMLSqueezer

It seems that -apart of the KEGGconverter- the use of KEGG2SBML tool is so far, the only other functional solution for the SBML conversion task. However, this solution requires a UNIX-based operational environment, together with Perl5 and KEGG database files, as previously mentioned. The execution of the conversion is performed through the assignment from the command line of a command like: "kegg2sbml -l 2 -v 1 -c 0 ./PATHWAY/hsa/hsa00010_cpd.coord", which produces a corresponding SBML/hsa/hsa00010.xml file. The switches shown in the specific command are for the production of Level 2 Version 1 SBML files without CellDesigner annotations.

At the next step the converted files were inserted in the semanticSBML tool [20] (version 1 beta). This application completed the merging process and produced an integrated SBML Level 2 Version 3 model.

The installation of semanticSBML entails the pre-installation of other packages such as: Python, Qt4, PyQt3, SOAP.py, libSBML3[21], Graphviz (currently not working in MS Windows but only required for the view function). There is also an online version of this tool, which produced the merged SBML file after the uploading of the six SBML models.

**Figure 7:**
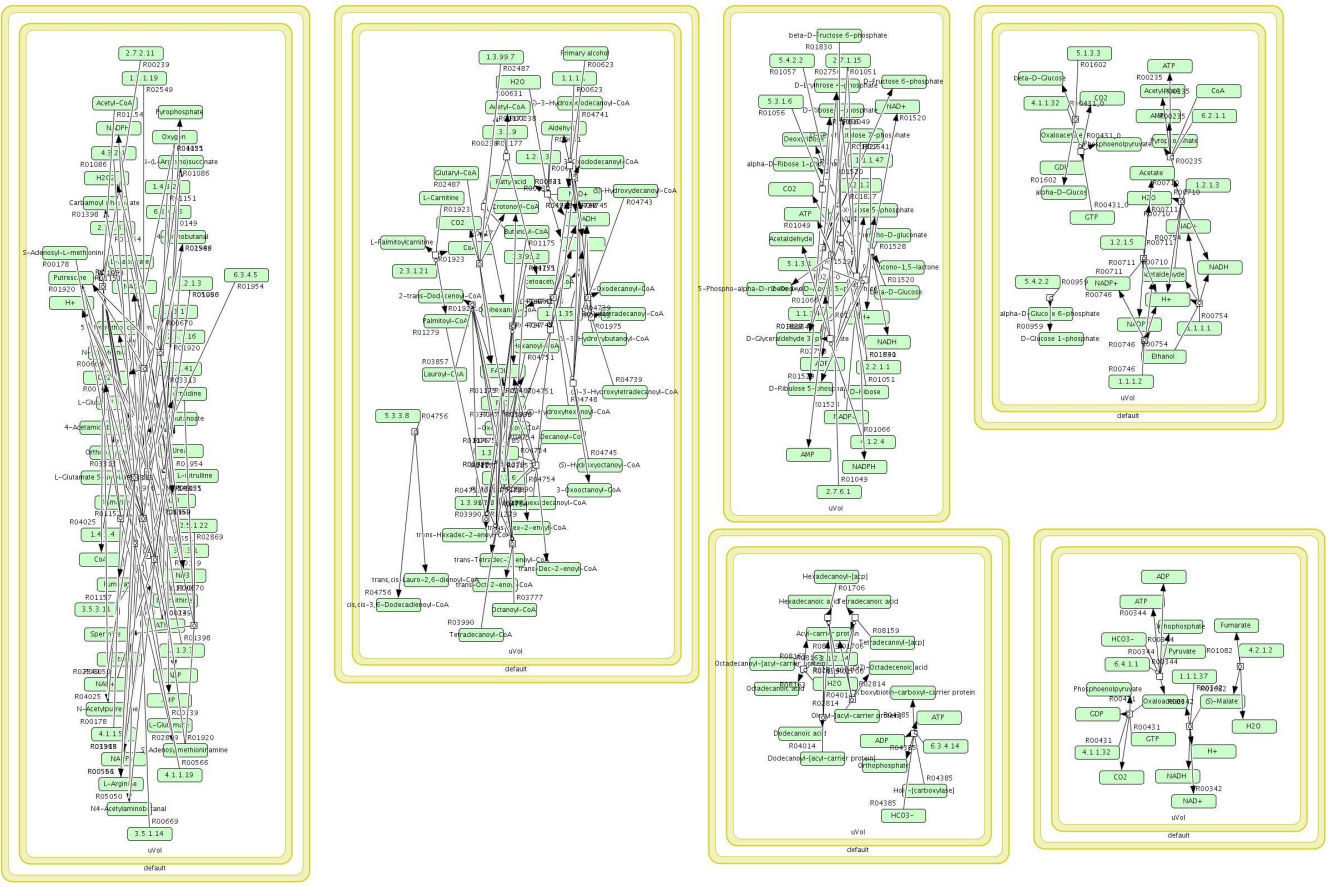
**Alternative procedure: KEGG2SBML → SemanticSBML**. Compartmental division of the pathways. The model contained 6 different compartments, one for each of the case pathways.

**Figure 8:**
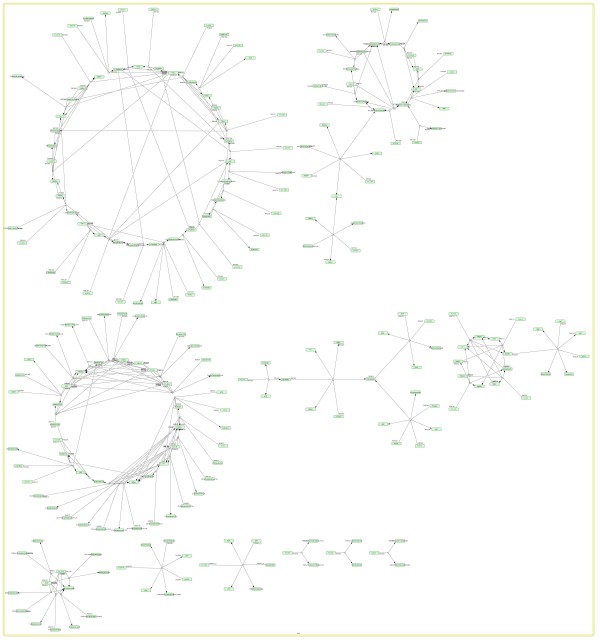
**Alternative procedure: KEGG2SBML → SemanticSBML**. Unconnected pathways. Even after the manual removal of different compartments, the pathways do not integrate.

The inspection of the L2V3 SBML model became possible in CellDesigner 4.0.1 only after the conversion of the model (using Python and libSBML3) to a lower version (L2V1), since L2V3 SBML is not yet supported in CellDesigner. Scrutinizing the derived model, problems were revealed, introduced by the semanticSBML tool, during the SBML conversion of KEGG pathways: the model contained 6 different compartments, one for each of our case pathways (Figure 7). Even after intensive manual editing of the file, for the elimination of redundant compartments, the integrated pathway could not be reproduced, as without the duplicates elimination procedure, the pathways remain separate, as seen in Figure 8.

An alternative approach for the merging of the six SBML models was made possible by running the simple bash script "mergeSBML.sh" mentioned in [22]. This script produces a merged SBML model which successfully integrates the imported pathways.

Nevertheless, a comparison to the number of relations included in the two integrated models, shows that the model which was constructed with the alternative procedure (KEGG2SBML → SemanticSBML/mergeSBML) contained 70 distinct reactions, while the model from KEGGconverter incorporated 154 reactions, not counting those that refer to the boundary conditions to neighbouring pathways. This radical difference can be attributed to the significantly less information that KEGG2SBML manages to extract from the KEGG flat files, comparing to the KGML files. This difference, of course, seriously deteriorates, as the number of the merged pathways increases.

The last step before the simulation phase for our test case is the insertion of kinetic information about the pertaining reactions of the model, through the tool SBMLsqueezer[23]. This tool has the ability to massively implement the incorporation of reaction kinetics where missing, by selecting some default values. In this way, it renders the model ready for the simulation phase. It works as a CellDesigner plug-in, and its installation entails only the downloading of the jar file in the plug-in directory of CellDesigner.

Currently, KEGGconverter does not add SBML annotations concerning the involved species and reactions, whereas SemanticSBML provide for a MIRIAM [24]-complied data format and SBMLsqueezer incorporates annotations in the form of Systems Biology Ontology (SBO) [25] [See additional file 3: for a comparison of features table relating the case study tools].

## Conclusion

KEGGconverter is capable of producing integrated metabolic pathway models ready for simulation purposes taking as input KEGG KGML files. It handles automatically transparently and seamlessly the fusion, conversion to SBML, proper renaming of the species, stoichiometrical correction of the integrated pathway and insertion of reaction specific default kinetic laws, in a straightforward manner compared to other solutions mentioned in the case study.

The final models thus derived, do not enclose trivial metabolites as they do not reproduce inconsistencies of the KGML visualization-oriented, simplified information pattern. At the same time they can be considered as information rich for dynamic in-silico simulations as they contain all the available information regarding the number the stoichiometry and the putative kinetic properties of the included reactions in each pathway. Furthermore, additional reactions to neighbouring pathways are constructed which indicate the direction of the metabolic flows in the network and thus providing better stability in the boundary conditions of the models.

## Availability and requirements

The current version of KEGGconverter is contained within this article (see Additional file 4 - KEGGconverter.jar), and is also available through the project homepage (where program related updates are posted too).

Project name: KEGGconverter

Project homepage: 

Operating Systems: Platform independent; tested on Windows and Linux.

Programming Language: Java

Hardware requirements: A machine with at least 512 MB of RAM but in the case of converting CellDesigner annotated files at least 1 GB is highly recommended. If memory issues arise during runtime, increasing maximum heap size using the -Xmx option of java is recommended.

Other Requirements: Java 6, libSBML 2.3.4 installed (for the command line case) is necessary for kinetics implementation.

License: Creative Commons Attribution-Noncommercial-Share Alike 3.0 Greece License 

## Conflict of interests

The authors declare that they have no competing interests.

## Authors' contributions

KM co-drafted the manuscript, co-programmed the application and carried out the case study. IK co-programmed the application and co-drafted the manuscript. AC conceived and designed the application, tested and evaluated the application, co-drafted the manuscript and supervised the implementation of the computational part of this work. IM provided significant corrections to the implementation of the code. FNK revised the manuscript and supervised the work. All authors read and approved the final manuscript.

## Supplementary Material

Additional file 1 **Command line options**. File containing the description of the commands of the command line version of KEGGConverter. Click here for file

Additional file 2 **Case study files**. **Input**: 6 KGML Pathway files: Glycolysis/Gluconeogenesis - hsa00010.xml, Citrate cycle (TCA cycle) - hsa00020.xml, Pentose phosphate pathway - hsa00030.xml, Fatty acid biosynthesis - hsa00061.xml, Fatty acid metabolism - hsa00071.xml, Urea cycle and metabolism of amino groups - hsa00220.xml. **Output from KEGGconverter**: the initial merged KGML file: mergedKEGG.xml, SBML converted model: sbml_mergedKEGGconverter.xml, SBML converted model with default kinetics: sbml_mergedKEGGconverterKinetics.xml, a circular layout diagram of the resulted model from CellDesigner: CaseStudyFinalDiagram.pdf. Click here for file

Additional file 3 **Case study files with alternative procedures**. • FeaturesComparisonTable.doc: a comparison of features table regarding the applications mentioned in the case study. • hsa_paths.zip: The 6 case study pathways produced by KEGG2SBML. • RamanAllMerged.xml: The intergrated SBML model using [22] alternative procedure and having as input the 6 SBML files produced by KEGG2SBML. • semanticAll.xml: The intergrated SBML model using semanticSBML and having as input the 6 SBML files produced by KEGG2SBML. Click here for file

Additional file 4 **KEGGconverter tool**. This is the version (1.0) of the KEGGconverter tool which runs as a command line utility. It searches in the '/in' subdirectory for KGML models and performs the conversion depending on the specific command arguments. Click here for file
