# Supplementary material for: KEGGconverter: a tool for the in-silico modelling of metabolic networks of the KEGG Pathways database
Source: BMC Bioinformatics. 2009 Oct 8;10:324. doi: 10.1186/1471-2105-10-324 (PMC2764712; doi:10.1186/1471-2105-10-324)
Supplement: Additional file 1 — Command line options. File containing the description of the commands of the command line version of KEGGConverter. [file 1471-2105-10-324-S1.DOC]

**Usage:**

In command line window type: *'java -jar KeggConvert.jar'*.

**Available options:**

*'justConvert'* to process kgml files from .\in directory.

*'keepOrphan'* to prevent orphan node elimination during model curation. Used together with the ‘*justConvert’* and ‘*justConvertCD’* options.

*'justConvertCD'* to process kgml files from .\in directory to CellDesigner sbml.

*'makeKinetics'* to convert and introduce kinetics in the produced sbml files.

*'makeKineticsCD'* to convert and introduce kinetics in the produced CellDesigner

sbml files.

*'merge'* to produce a merged kgml file for all KEGG files in .\in directory.

*'STATS'* to produce reaction statistics for all sbml files in .\out directory.

*'dir=mydir/'* to use as subdirectories in in&out directories.

*'help'* to get these available options.

In case of being behind proxies you must use options before '-jar':

'*-Dhttp.proxyHost=xxx.xx.xxx.x -Dhttp.proxyPort=8080*'
