# Supplementary figures and images for: KEGGconverter: a tool for the in-silico modelling of metabolic networks of the KEGG Pathways database
Source: BMC Bioinformatics. 2009 Oct 8;10:324. doi: 10.1186/1471-2105-10-324 (PMC2764712; doi:10.1186/1471-2105-10-324)

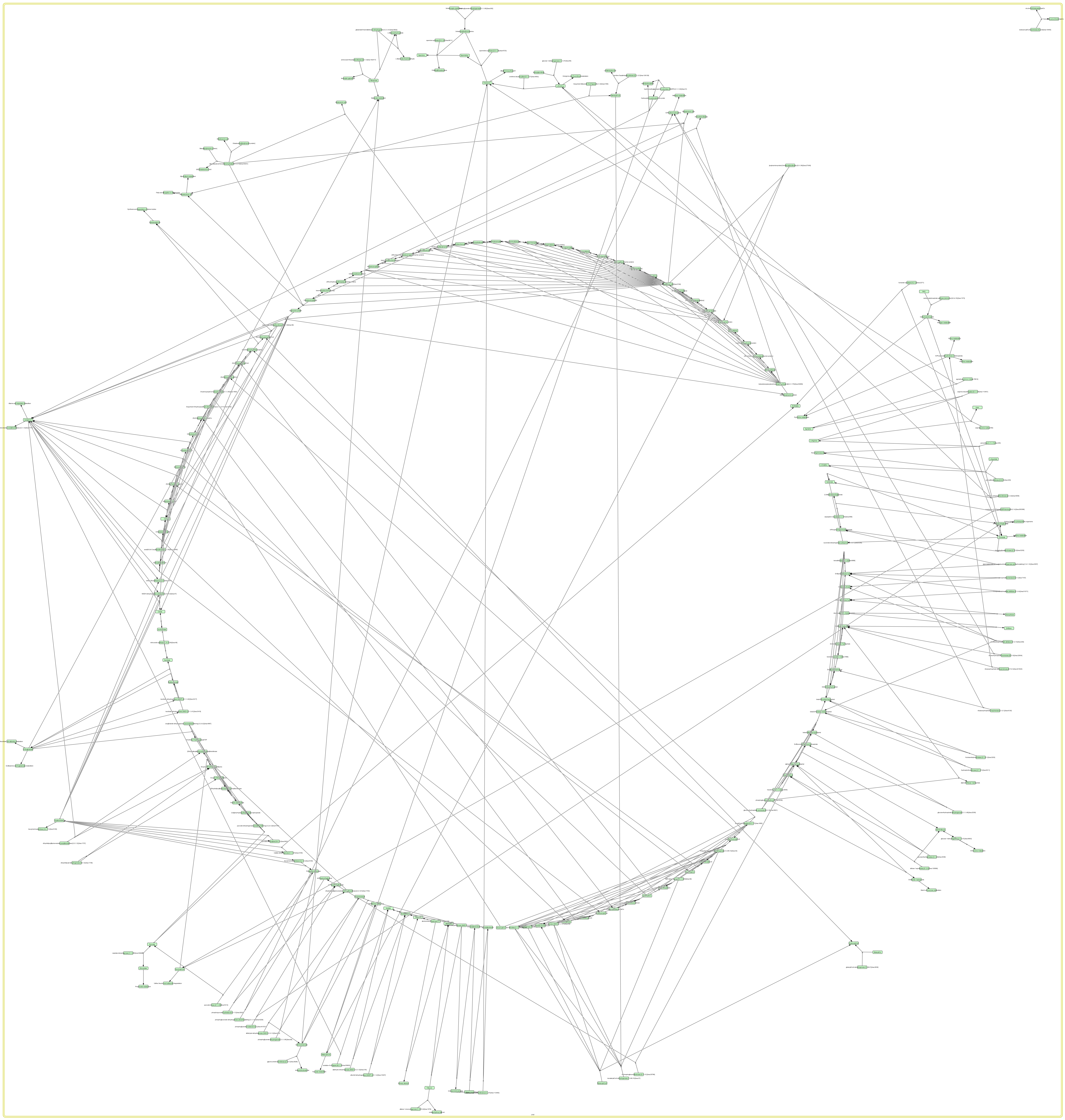

Supplement: Additional file 2 — Case study files. Input: 6 KGML Pathway files: Glycolysis/Gluconeogenesis - hsa00010.xml, Citrate cycle (TCA cycle) - hsa00020.xml, Pentose phosphate pathway - hsa00030.xml, Fatty acid biosynthesis - hsa00061.xml, Fatty acid metabolism - hsa00071.xml, Urea cycle and metabolism of amino groups - hsa00220.xml. Output from KEGGconverter: the initial merged KGML file: mergedKEGG.xml, SBML converted model: sbml_mergedKEGGconverter.xml, SBML converted model with default kinetics: sbml_mergedKEGGconverterKinetics.xml, a circular layout diagram of the resulted model from CellDesigner: CaseStudyFinalDiagram.pdf. [file 1471-2105-10-324-S2.ZIP › File1/CaseStudyFinalDiagram.pdf]
