# Supplementary material for: KEGGconverter: a tool for the in-silico modelling of metabolic networks of the KEGG Pathways database
Source: BMC Bioinformatics. 2009 Oct 8;10:324. doi: 10.1186/1471-2105-10-324 (PMC2764712; doi:10.1186/1471-2105-10-324)
Supplement: Additional file 3 — Case study files with alternative procedures. • FeaturesComparisonTable.doc: a comparison of features table regarding the applications mentioned in the case study. • hsa_paths.zip: The 6 case study pathways produced by KEGG2SBML. • RamanAllMerged.xml: The intergrated SBML model using [22] alternative procedure and having as input the 6 SBML files produced by KEGG2SBML. • semanticAll.xml: The intergrated SBML model using semanticSBML and having as input the 6 SBML files produced by KEGG2SBML. [file 1471-2105-10-324-S3.ZIP › File2/FeaturesComparisonTable.doc]

|  | **Reads KGML** | **Converts to SBML** | **Merging** | **Redundancy** | **Removes Duplicates** | **Graph editing** | **Kinetics** | **Annotations** | **Environment** |
| --- | --- | --- | --- | --- | --- | --- | --- | --- | --- |
| **KGML-ED** | yes |  | yes | yes (manually) | yes | yes |  |  | independent |
| **KEGG2SBML** | No, it uses KEGG DB files | yes |  |  |  |  |  |  | Unix like |
| **semanticSBML** |  | yes  L2V3 | yes | yes | yes | yes (for simple graphs) |  | yes | Windows / Linux & on-line |
| **SBMLsqueezer** |  |  |  |  |  |  | yes | yes | CellDesigner |
| **KEGGconverter** | yes | yes | yes | yes | yes |  | yes |  | independent |
